# Arginase-1 inhibition reduces migration ability and metastatic colonization of colon cancer cells

**DOI:** 10.1186/s40170-022-00301-z

**Published:** 2023-01-13

**Authors:** Xiangdong Wang, Huihui Xiang, Yujiro Toyoshima, Weidong Shen, Shunsuke Shichi, Hiroki Nakamoto, Saori Kimura, Ko Sugiyama, Shigenori Homma, Yohei Miyagi, Akinobu Taketomi, Hidemitsu Kitamura

**Affiliations:** 1 Division of Functional Immunology, Section of Disease Control, Institute for Genetic Medicine, Hokkaido University, Kita-15, Nishi-7, Kita-ku, Sapporo, 060-0815 Japan; 2 Molecular Pathology and Genetics Division, Kanagawa Cancer Center Research Institute, Yokohama, 241-8515 Japan; 3 Department of Gastroenterological Surgery I, Hokkaido University Graduate School of Medicine, Sapporo, 060-8638 Japan

**Keywords:** Arginase 1, Arginine, Metastatic colonization, Malignancy, Colorectal cancer

## Abstract

**Background:**

Arginase-1 (ARG1), a urea cycle-related enzyme, catalyzes the hydrolysis of arginine to urea and ornithine, which regulates the proliferation, differentiation, and function of various cells. However, it is unclear whether ARG1 controls the progression and malignant alterations of colon cancer.

**Methods:**

We established metastatic colonization mouse model and ARG1 overexpressing murine colon cancer CT26 cells to investigate whether activation of ARG1 was related to malignancy of colon cancer cells in vivo. Living cell numbers and migration ability of CT26 cells were evaluated in the presence of ARG inhibitor in vitro.

**Results:**

Inhibition of arginase activity significantly suppressed the proliferation and migration ability of CT26 murine colon cancer cells in vitro. Overexpression of ARG1 in CT26 cells reduced intracellular l-arginine levels, enhanced cell migration, and promoted epithelial-mesenchymal transition. Metastatic colonization of CT26 cells in lung and liver tissues was significantly augmented by ARG1 overexpression in vivo. ARG1 gene expression was higher in the tumor tissues of liver metastasis than those of primary tumor, and arginase inhibition suppressed the migration ability of HCT116 human colon cancer cells.

**Conclusion:**

Activation of ARG1 is related to the migration ability and metastatic colonization of colon cancer cells, and blockade of this process may be a novel strategy for controlling cancer malignancy.

**Supplementary Information:**

The online version contains supplementary material available at 10.1186/s40170-022-00301-z.

## Introduction

Colorectal cancer (CRC) is the third most frequently diagnosed cancer type worldwide [1]. Epidemiological data show that CRC is projected to increase by 60% to more than 2.2 million new cases and 1.1 million deaths by 2030 [2]. The deaths caused by tumor recurrence and metastasis account for most of the causes of death in all CRC patients [3]. Current treatment outcomes for primary CRC have improved, but metastases to other organs such as the liver, lung, and peritoneum remain difficult to cure despite the development of surgery, chemotherapy, and targeted therapies. Therefore, investigating malignant factors in the process of recurrence and metastasis is crucial for the regulation of CRC. Generally, CRC is a multistage carcinogenesis that leads to cancer through the appearance of adenoma and is accompanied by some genetic abnormalities [4], but the multistage carcinogenesis theory predicts the existence of further metastasis-related genetic abnormalities. Despite widespread research to compare primary and metastatic lesions, no genetic abnormality governing metastasis has yet been identified.

Amino acids have critical functions in protein synthesis, providing intermediate metabolites and mediating relevant biological processes [5]. Previous research demonstrated that cancer progression occurs through the metabolism of branched-chain amino acids in myeloid leukemia [6], and a shift in glutamine nitrogen metabolism contributes to the malignant progression of cancer [7]. Alteration of amino acid transporters to support aspartate and glutamate dependency sustains endocrine resistance in breast cancer [8]. TGF-β-dependent reprogramming of amino acid metabolism induces epithelial-mesenchymal transition (EMT) in non-small cell lung cancer [9]. Recently, we discovered that IL-6 produced in the tumor microenvironment augmented arginase-1 (ARG1) activity, and blockade of ARG1 significantly reduced tumorigenesis in a tumor-bearing model [10].

ARG1, a urea cycle-related enzyme, catalyzes the hydrolysis of arginine to urea and ornithine, which regulates the proliferation, differentiation, and function of various cells, including immune cells [11–14]. Macrophage metabolism of apoptotic cell-derived arginine promotes continual efferocytosis and resolution of injury [15], and activation of ARG1 suppresses dendritic cell-mediated anti-tumor immunity [10, 16]. However, a previous study indicated that l-arginine had a significant difference in the metabolism of normal and malignant cells [17]. ARG1 is closely related to collagen synthesis and bioenergy, which are critical for malignant cell proliferation and invasiveness [18–20]. However, whether ARG1 activation is related to the progression and metastatic colonization of colon cancer cells remains unclear.

In this study, we found that inhibition of arginase activity significantly suppressed the migration ability of colon cancer cells. Overexpression of ARG1 in colon cancer cells reduced their intracellular l-arginine levels, enhancing their migration ability. Finally, we confirmed that *Arg1* gene expression was higher in tumor tissues of liver metastasis than in those of the primary tumor of CRC patients. We report here that ARG1 activation is involved in the metastatic colonization of colon cancer cells and its blockade may be a novel strategy for cancer malignancy.

## Materials and methods

### Antibodies and reagents

FITC-conjugated Annexin V and PE-Cyanine7-conjugated anti-CD45 antibody were obtained from BioLegend (San Diego, CA, USA). 7-Amino-actinomycin D (7AAD) was purchased from Beckman Coulter (Brea, CA, USA). Anti-ARG1 (HPA024006) and anti-α-tubulin (DM1A) antibodies were purchased from Sigma-Aldrich (St. Louis, MO, USA). Anti-N-cadherin (sc-7939) antibody was purchased from Santa Cruz Biotechnology (Dallas, TX, USA). Anti-E-cadherin (AF748) antibody was purchased from R&D Systems (Minneapolis, MN, USA). An ARG1 antagonist (nor-NOHA) was purchased from Enzo Biochem (New York, NY, USA).

### Mice and cell lines

Wild-type BALB/c mice were purchased from Charles River Japan (Kanagawa, Japan). All mice were maintained in specific pathogen-free conditions in accordance with the guidelines of the Animal Department at Hokkaido University and were used at 6–8 weeks of age. All mouse experiments were approved by the Animal Ethics Committee of Hokkaido University (19-0036, 21-0026) and conducted in accordance with the recommendations of the Guide for the Care and Use of Laboratory Animals of the University, an Institutional Animal Care and Use Committee.

A murine colon carcinoma cell line, CT26 (CRL-2638), was obtained from the American Type Culture Collection (Manassas, VA, USA) in 2013. The cell lines used in the experiments were cultured for a maximum of 20 passages before use. CT26 cells were maintained in RPMI-1640 medium (Wako Pure Chemical Industries) supplemented with 10% fetal bovine serum (#172012; Nichirei Bioscience), penicillin (200 U/mL), streptomycin (100 μg/mL; Meiji Seika Pharma), 10 mM HEPES (Wako Pure Chemical Industries), and 2-mercaptoethanol (0.05 mmol/L; Sigma-Aldrich, Tokyo, Japan) at 37 °C in a humidified atmosphere containing 5% CO_2_. CT26 mock control cells (mock control) and ARG1-overexpressing cells (*Arg1* OE) were established by transfection with pMX-IRES-GFP obtained from The University of Tokyo using Lipofectamine 3000 (Thermo Fisher Scientific, Waltham, MA, USA) in accordance with the manufacturer’s protocol.

### Human subjects

Research protocols involving human subjects were approved by the institutional review board of Hokkaido University Graduate School of Medicine (14-042, 14-043) and the Institute for Genetic Medicine (14-0003, 14-0004). Written informed consent was obtained from 11 healthy donors and 10 CRC patients, who underwent a surgical operation at Hokkaido University Hospital between 2003 and 2015 and were included in this study.

### Real-time PCR

Total RNA was extracted from culture cells or isolated GPF^+^CD45^−^ cells from CT26-bearing mice using ISOGEN (311-07361; Nippon Gene) in accordance with the manufacturer’s instructions. RNA concentration was measured using a NanoDrop Spectrophotometer (#ND-1000; Thermo Fisher Scientific). First-strand cDNA was synthesized using 1 μg total RNA and ReverTra Ace qPCR RT Master Mix (TOYOBO, Japan) and then amplified with a thermal cycler (Veriti; Applied Biosystems). The template DNA was used for a final PCR reaction volume of 10 μL. Genes encoding murine *Arg1* and *Actb* were amplified and detected using THUNDERBIRD SYBR qPCR Mix (TOYOBO) and StepOnePlus (Applied Biosystems). The primer sequences used in this study were as follows: *Arg1* (left: 5′-cctgaaggaactgaaaggaaag-3′, right: 5′-ttggcagatatgcagggagt-3′), *Twist1* (left: 5′-agctacgccttctccgtct-3′, right: 5′-tccttctctggaaacaargaca-3′), *Twist2* (left: 5′-catgtccgcctcccacta-3′, right: 5′-gatgtgcaggtgggtcct-3′), *Zeb2* (left: 5′-ccagaggaaacaaggatttcag-3′, right: 5′-aggcctgacatgtagtcttgtg-3′), and *Actb* (left: 5′-aaggccaaccgtgaaaagat-3′, right: 5′-gtggtacgaccagaggcatac-3′). Sample signals were normalized to the reference gene *Actb* using the ^ΔΔ^Ct method: ^Δ^Ct = ^Δ^Ct_sample_ − ^Δ^Ct_reference_. Percentages relative to the control sample were then calculated for each sample.

### Western blotting

Cell lysates were prepared from CT26 cells using cold RIPA buffer (Thermo Fisher Scientific) supplemented with protease inhibitor cocktail (539131; Sigma-Aldrich) and phosphatase inhibitor (07574-61; Nacalai Tesque). Samples were separated by SDS-PAGE and transferred to PVDF membranes (Millipore, Billerica, MA, USA). The membranes were probed with primary antibodies at 4 °C overnight, followed by incubation with HRP-conjugated anti-rabbit IgG (398548; GE Healthcare) or mouse IgG (7076; Cell Signaling Technology) secondary antibody at room temperature for 2 h following the manufacturer’s protocol. The protein signals were detected with ECL Prime Western Blotting Detection Reagents (RPN2232; Amersham, UK) and visualized using an Image Quant LAS4000 mini (GE Healthcare, USA). The N-cadherin and E-cadherin signals were standardized based on those of α-tubulin, using the ImageJ software (National Institutes of Health).

### Living cell number assay

CT26 (2 × 10^3^) and HCT116 (5 × 10^3^) cells were cultured with or without ARG1 inhibitor, nor-NOHA (0, 125, 250, and 500 μM), in 96-well plates and incubated at 37 °C for 12 h or 24 h. Living cell numbers were determined using a Cell Counting Kit-8 (CCK-8, Dojindo Molecular Technologies, Kumamoto, Japan) as per the manufacturer’s protocol. The absorbance (450 nm) was measured by absorption spectrometry (EZS-ABS; IWAKI, Tokyo, Japan) and indicated as an index of living cell numbers.

### Flow cytometry

CT26 cells were cultured with or without ARG1 inhibitor, nor-NOHA (0, 125, 250, and 500 μM), in 96-well plates and incubated at 37 °C for 12 h or 24 h. The expression levels of Annexin V and 7AAD were evaluated by FACSCanto II (BD Biosciences), and the percentage of CT26 apoptotic cells was analyzed with the FlowJo software (Tree Star, Ashland, OR, USA).

### Cell migration assay

Cell migration assays were performed using ThinCert Tissue Culture Inserts for Multiwell Plates following the protocol (8 um pore size, 24 well; Greiner Bio-one). CT26 (1 × 10^4^) or HCT116 (2 × 10^4^) cells were inoculated in the upper chamber with serum-free medium. Medium containing 20% fetal bovine serum was added to the lower chamber as a chemoattractant. CT26 or HCT116 (2 × 10^4^) cells were incubated at 37 °C in a humidified atmosphere containing 5% CO_2_ for 24 h. After the cells on the upper surface of the insert were removed, the cells that had migrated to the bottom of the insert were stained with hematoxylin and eosin (HE). The stained cells were counted at four different microscopic fields, and the migration ability was evaluated from the numbers.

### Arginase activity assay

Arginase activity was measured as follows. After preincubation for 24 h, CT26 cells (1 × 10^6^) were re-suspended in 10 mM Tris-HCL (100 μL, pH 7.4) with 0.4% Triton X-100 and protease inhibitor cocktail (Nacalai Tesque). Arginase activity in each sample (40 μL) was determined by Quantichrom Arginase Assay Kit (BioAssay Systems) in accordance with the manufacturer’s protocol. Briefly, all reagents were brought to room temperature prior to assay. Urea standard solution diluted with distilled water (1 mM) and 5 × Substrate Buffer contained with arginine and Mn was adjusted immediately prior to each test. Serum samples (40 μL) were mixed with or without 5 × Substrate Buffer (10 μL) in a 96-well plate and incubated at 37 °C for 2 h. Urea reagent (200 μL) was prepared by combining equal volumes of reagent A and reagent B and added to all wells. The optical density at 430 nm was measured by absorption spectrometry (EZS-ABS; IWAKI, Tokyo, Japan). Arginase activity was calculated as follows: arginase = (OD_sample_ − OD_blank_)/(OD_standard_ − OD _water_) × 10.4 (U/L). In some experiments, CT26 mock control or ARG1-overexpressing cells (1 × 10^6^) were re-suspended in 10 mM Tris-HCL (100 μL, pH 7.4) and lysed with 0.4% Triton X-100 in the presence of protease inhibitor cocktail (Nacalai Tesque, Osaka, Japan). The cell lysate samples (40 μL) were used for the assay.

### Measurement of free amino acids

The serum (100 μL) collected from mice was diluted twice with 10% trichloroacetic acid and centrifuged at 9730×*g* for 15 min. The supernatants were collected and processed in 0.22 μM SPIN-X Centrifuge Tube Filters (Corning Costar) and used for the analyses. CT26 mock control cells and *Arg1* OE cells (5 × 10^6^) were cultured in 100-mm cell culture dishes for 24 h. After washing with PBS, the cells were collected and centrifuged at 2430×*g* for 5 min. Methanol (1 mL) was added to the cell pellets and incubated at room temperature for 10 min, and then H_2_O (0.5 mL) and chloroform were further added before centrifugation at 21,880×*g* for 15 min. The supernatants were collected and processed with 10-kDa centrifugal filter units (Millipore) and used for analysis. The prepared samples were analyzed with an amino acid analyzer (L-8900; Hitachi High-Technologies Corporation, Tokyo, Japan) in the Global Facility Center at Hokkaido University.

### Metastatic colonization model

GFP-transfected CT26 mock control cells and *Arg1* OE cells (2 × 10^5^) were inoculated intrasplenically or intravenously into wild-type BALB/c mice as previously described [21], and the liver and lung tissues were collected on day 14. Metastatic colonization images of CT26-GFP^+^ tumors were calculated using epi-fluorescence on an IVIS spectrum ex vivo imaging system (Xenogen). In some experiments, l-arginine (500 mg/kg) or nor-NOHA (20 mg/kg), an arginase inhibitor, was intraperitoneally injected every 2 days. Then, a 4% paraformaldehyde phosphate buffer solution (Wako Pure Chemical Industries, Osaka, Japan) was used to fix the collected tissue, which was embedded in paraffin blocks that were serially cut and stained by HE. In some experiments, CT26-GFP^+^CD45^−^ colon cancer cells were isolated from the liver tissues of metastatic colonization model mice by FACSAriaII (BD Bioscience) as previously described [21], and ARG1 gene expression levels of the isolated cells were evaluated by real-time PCR.

### Public datasets

The dataset with ARG1 protein expression from patients with colorectal cancer was downloaded from Clinical Proteomic Tumor Analysis Consortium (CPTAC, PDC000116) (https://proteomic.datacommons.cancer.gov/pdc/browse/filters/primary_site:Colon%7CRectum). One hundred normal colon tissue samples were evaluated in the Tandem Mass Tag (TMT) 10-plex, with 96 normal samples paired with tumor samples from the same participant. The 4 remaining normal colon tissue samples from the Pacific Northwest National Laboratory (PNNL) dataset pair with 4 tumor tissue samples assayed by Vanderbilt University Medical Center (VUMC). In total, these data include tumor and normal global proteomic profiling from 100 individuals. Relative protein abundance was calculated as the ratio of sample abundance to reference abundance using the summed reporter ion intensities from peptides that could be uniquely mapped to a gene. The pooled reference sample was labeled with TMT 131 reagent, allowing a comparison of relative protein abundances across different TMT-10 plexes. The relative abundances were log2-transformed and zero-centered for each gene to obtain final, relative abundance values. Finally, after filtering the missing values of ARG1 protein abundance samples, we obtained ARG1 protein expression data in normal (*N* = 28) and tumor (*N* = 42) tissues of CRC patients.

Microarray gene expression profiles of 18 liver metastasis (LM) matched with primary colorectal cancer (pCRC) in the same patient samples in the GSE14297 dataset were downloaded from the Gene Expression Omnibus database (https://www.ncbi.nlm.nih.gov/geo/query/acc.cgi?acc=GSE14297), using the “GEOquery” Bioconductor R package (v2.62.2) (https://bioconductor.org/packages/release/bioc/html/GEOquery.html).

### Statistical analysis

In vitro experiments were repeated 3–6 times. In vivo experiments consisting of 5–8 mice per group were independently performed 2–3 times. The mean values and standard deviations (SDs) were calculated for each dataset. Significant differences in the results were determined by the two-tailed Student’s *t*-test. *P*-values < 0.05 were considered statistically significant. In some experiments, data were analyzed using the JMP statistical software for Windows (version 13.1.0; SAS Institute Inc., Cary, NC, USA).

## Results

### l-arginine and arginase activity are related to metastatic colonization of colon cancer cells in the liver

**Fig. 1:**
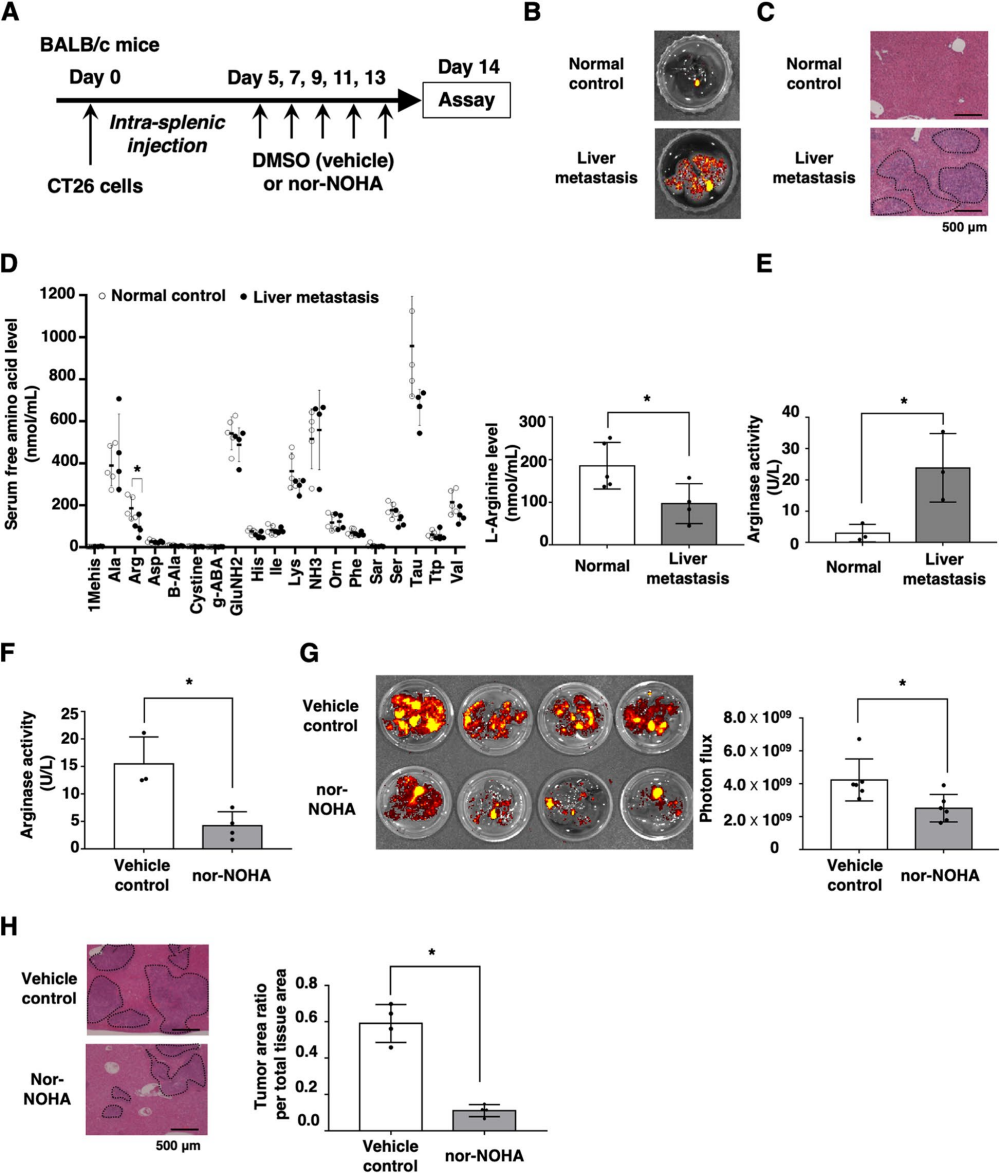
Effect of l-arginine and arginase activity on the metastatic colonization of colon cancer cells. GFP-transfected CT26 murine colon cancer cells (2 × 10^5^) were intrasplenically inoculated into wild-type BALB/c mice (day 0). Then, nor-NOHA (20 mg/kg) was injected intraperitoneally on days 5, 7, 9, 11, and 13. **A** Experimental schemes are shown. **B** Metastatic colonization in liver tissue was evaluated using an in vivo imaging system on day 14. Representative images of normal liver and GFP-expressing CT26 cell-bearing livers are shown. **C** HE staining of liver tissues was performed, and representative images are shown. **D** The sera were collected from normal and liver metastatic colonization model mice on day 14. Serum-free amino acid levels were evaluated by HPLC. The mean values and SDs (*n* = 4–5, three independent experiments) are shown. **E** Serum arginase activities of normal and liver metastasis model mice were determined by EIA. The mean values and SDs (*n* = 4) are shown. **F** Serum arginase activities of liver metastasis model mice injected with DMSO or nor-NOHA (20 mg/kg) were determined by EIA. The mean values and SDs (*n* = 4) are shown. **G** Metastatic colonization in liver tissue of mice injected with DMSO or nor-NOHA (20 mg/kg) was evaluated using an in vivo imaging system on day 14. Representative images of normal liver and GFP-expressing CT26 cell-bearing livers are shown. Photon flux ratios were determined from images of liver metastatic colonization model mice (*n* = 4, three independent experiments). *P < 0.05 by Student’s *t*-test. **H** HE staining of liver tissue was performed 14 days after inoculation. Bars in the images represent 500 μm. Ratios of the tumor area relative to the total liver tissue area were calculated by the ImageJ software. The mean values and SDs from four independent mice are shown. *P < 0.05 by Student’s *t*-test

To evaluate the effects of arginase activation on the malignant alterations of colon cancer cells, we established a liver metastatic colonization model through intrasplenic injection of GFP-transduced CT26 murine colon cancer cells into wild-type BALB/c mice, followed by treatment with nor-NOHA, an arginase inhibitor (Fig. 1A). In vivo imaging analysis and HE staining demonstrated the tumorigenesis of CT26 cells in liver tissues (Fig. 1B, C). High-performance liquid chromatography (HPLC) analysis of free amino acids revealed that serum l-arginine levels in the liver metastatic colonization model were significantly lower compared with those of the normal control, which were not injected with CT26 cells (Fig. 1D). Furthermore, the serum arginase activity of the liver metastatic model was higher compared with the normal control (Fig. 1E). Next, we performed intraperitoneal injection of nor-NOHA, an arginase inhibitor, into the liver metastatic colonization model to evaluate the effects of serum arginase enzymatic activity and arginine metabolism on the malignancy of colon cancer cells in vivo. Treatment with nor-NOHA significantly reduced serum arginase activity of the liver metastatic colonization model (Fig. 1F). Blockade of arginase activity significantly suppressed the liver metastatic colonization of CT26 cells in vivo (Fig. 1G, H). Furthermore, we performed the supplementation of l-arginine in the mouse model. As a result, we found that l-arginine supplementation significantly reduced metastatic colonization of CT26 cells in the liver of the mice (Additional file 1: Fig. S1). These data suggest that arginase-mediated arginine metabolism may be related to the metastatic colonization of colon cancer cells in the liver.

### Arginase activity regulates the migration ability of colon cancer cells in vitro

**Fig. 2:**
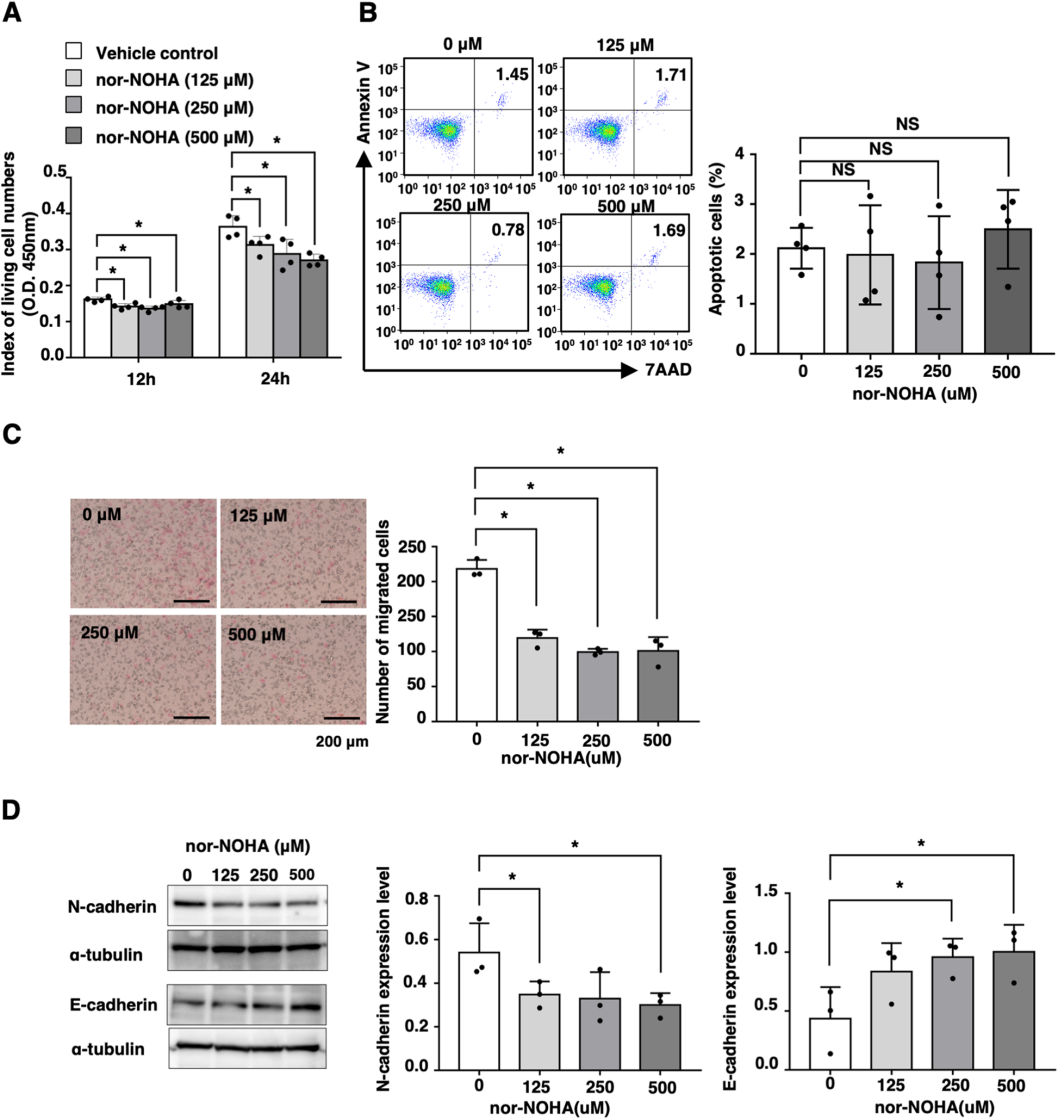
Arginase activity is related to intracellular l-arginine levels and the migration ability of colon cancer cells in vitro. **A** CT26 cells (2 × 10^3^) were cultured in the absence and presence of nor-NOHA (0, 125, 250, 500 μM). Living cell numbers were evaluated at 12 h and 24 h. The mean values and SDs (*n* = 4) are indicated. *P < 0.05 by Student’s *t*-test. **B** Apoptosis was evaluated by flow cytometry using 7AAD and Annexin V staining. Representative profiles are indicated. The percentage of apoptotic cells was calculated, and the mean values and SDs (*n* = 4) of the data are indicated. *P < 0.05 by Student’s *t*-test. **C** CT26 cells (1 × 10^4^) were cultured in the absence and presence of nor-NOHA (0, 125, 250, 500 μM). Migration ability was evaluated by Transwell assay at 24 h. Representative images are indicated. Bars represent 200 μM. The mean values and SDs (*n* = 4) of the data are indicated. *P < 0.05 by Student’s *t*-test. **D** N-cadherin and E-cadherin protein expression levels were determined by Western blotting. Representative images are indicated. The mean values and SDs (*n* = 3) of relative expression levels against α-tubulin are indicated. *P < 0.05 by Student’s *t*-test

Next, we investigated the effects of arginase activity on the malignant alterations of colon cancer cells in vitro. Treatment with nor-NOHA significantly suppressed the living cell numbers of CT26 cells in vitro in a dose-dependent manner (Fig. 2A), whereas the ratios of Annexin V- and/or 7AAD-positive cells were not altered by the addition of nor-NOHA (Fig. 2B). Cell migration assay revealed that inhibition of arginase activity significantly reduced the migration ability of CT26 cells in vitro (Fig. 2C). Then, we examined the effect of arginase activity on EMT. N-cadherin expression was downregulated, and E-cadherin expression was enhanced in nor-NOHA-treated CT26 cells (Fig. 2D). These findings suggest that arginase activity is involved in the migration as well as living cell numbers of colon cancer cells in vitro.

### ARG1 overexpression reduces intracellular l-arginine levels and augments the migration ability of colon cancer cells in vitro

**Fig. 3:**
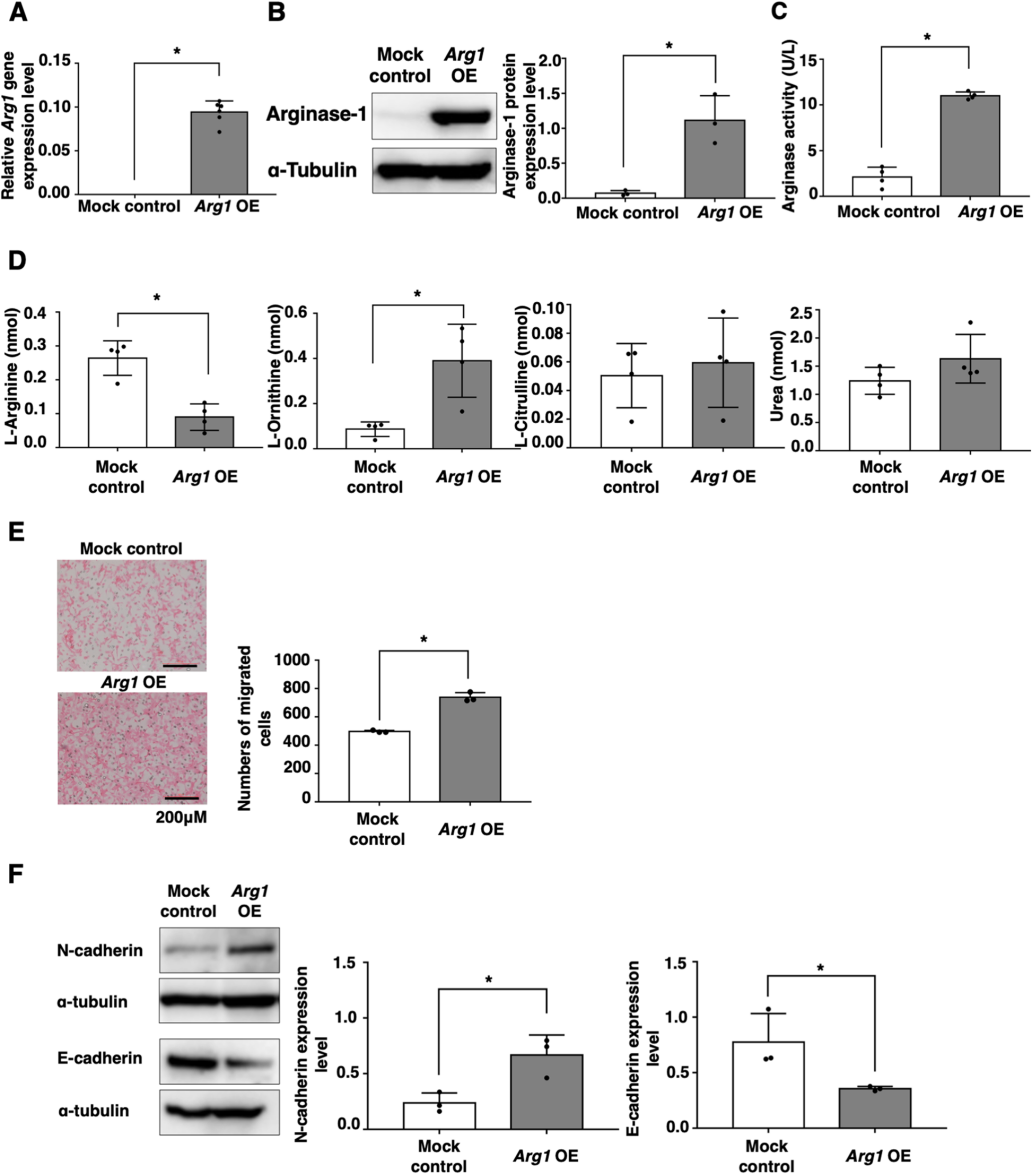
Overexpression of ARG1 augments EMT and the migration ability of cancer cells. GFP-transfected CT26 mock control and CT26 *Arg1* OE cells were established using the pMX-IRES-GFP vector. **A** Gene expression levels of *Arg1* were investigated by qPCR. The mean values and SDs (*n* = 4) are indicated. *P < 0.05 by Student’s *t*-test. **B** ARG1 protein expression levels were evaluated by Western blotting. The mean values and SDs (*n* = 3) are indicated. *P < 0.05 by Student’s *t*-test. **C** Intracellular ARG1 activity was determined by EIA. The mean values and SDs (*n* = 4) are shown. *P < 0.05 by Student’s *t*-test. **D** GFP-transfected CT26 mock control and CT26 *Arg1* OE cells (5 × 10^6^) were cultured for 24 h. Intracellular levels of l-Arginine, l-ornithine, l-citrulline, and urea were evaluated by HPLC, and the mean values and SDs (*n* = 4, three independent experiments) are shown. *P < 0.05 by Student’s *t*-test. **E** GFP-transfected CT26 mock control and CT26 *Arg1* OE cells (5 × 10^4^) were cultured for 24 h. Migration ability was evaluated by Transwell assay at 24 h. Representative images are indicated. Bars represent 200 μM. The mean values and SDs (*n* = 4) are indicated. *P < 0.05 by Student’s *t*-test. **F** N-cadherin and E-cadherin protein expression levels were determined by Western blotting. Representative images are indicated. The mean values and SDs (*n* = 3) of the relative expression levels against α-tubulin are indicated. *P < 0.05 by Student’s *t*-test

We established ARG1-overexpressing CT26 cells to evaluate the effects of ARG1-mediated arginine metabolism on the malignancy of colon cancer cells. *Arg1* was transfected into CT26 cells (*Arg1* OE) by the pMX vector system (Fig. 3A). We confirmed that ARG1 protein expression and the enzymatic activity of *Arg1* OE were significantly enhanced compared with the control cells (mock control) (Fig. 3B, C). To address the effects of ARG1 overexpression on amino acid metabolism in colon cancer cells, we conducted an analysis of the intracellular free amino acid levels for *Arg1* OE compared with the mock control. HPLC analysis showed that l-arginine levels of *Arg1* OE were significantly lower and l-ornithine levels were higher compared with the mock control, although l-citrulline and urea, urea cycle-related metabolites, were not altered (Fig. 3D). We further evaluated the effects of ARG1 overexpression on the malignant alteration of colon cancer cells. *Arg1* OE showed enhanced migration ability compared with the mock control in vitro (Fig. 3E). N-cadherin expression was enhanced and E-cadherin expression was reduced in *Arg1* OE compared with the mock control (Fig. 3F). We further evaluated the expression levels of EMT-related genes in *Arg1*-overexpressing CT26 cells. As a result, we found that *Twist1*, *Twist2*, and *Zeb2* gene expression levels were augmented in the *Arg1* OE CT26 cells compared to the mock control cells (Additional file 2: Fig. S2), suggesting that ARG1 overexpression caused EMT. These findings indicate that ARG1 activation may augment the malignant alteration of colon cancer cells.

### ARG1 overexpression in colon cancer cells augments their metastatic colonization ability in vivo

**Fig. 4:**
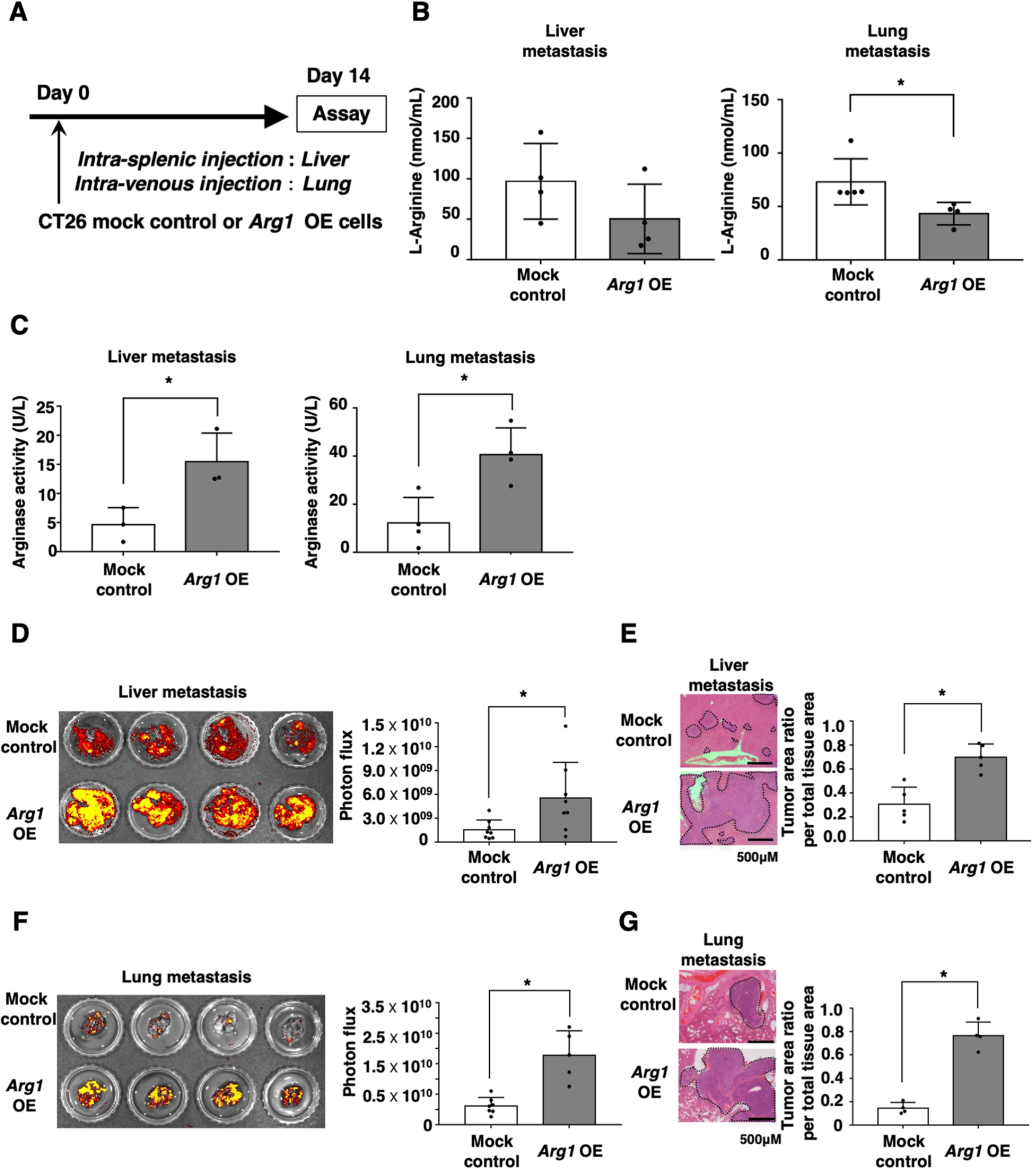
Overexpression of ARG1 augments the metastatic colonization ability of cancer cells. **A** GFP-transfected CT26 mock control and CT26 *Arg1* OE cells (2 × 10^5^) were intravenously or intrasplenically inoculated into mice. Sera and liver or lung tissues for assay were collected on day 14. **B** Serum l-arginine levels were evaluated by HPLC, and the mean values and SDs (*n* = 4, three independent experiments) are shown. **C** Serum arginase activity was determined by EIA, and the mean values and SDs (*n* = 4–5) are shown. **D** Metastatic colonization in the liver tissue of mice was evaluated using an in vivo imaging system on day 14. Representative images of normal liver and GFP-expressing CT26 cell-bearing livers are shown. Photon flux ratios were determined from the images of liver metastatic colonization model mice (*n* = 4, three independent experiments). **E** HE staining of liver tissue was performed 14 days after inoculation. Representative images are shown. Bars in the images represent 200 mm. **F** Metastatic colonization in the lung tissue of mice was evaluated using an in vivo imaging system at day 14. Representative images of normal liver and GFP-expressing CT26 cell-bearing livers are shown. Photon flux ratios were determined from images of the liver metastatic colonization model mice (*n* = 4, three independent experiments). **G** HE staining of lung tissue was performed 14 days after inoculation. Representative images are shown. Bars in the images represent 500 mm. Ratios of the tumor area relative to the total liver tissue area were calculated by the ImageJ software (**E**, **G**). The mean and SD values from four independent mice are shown

To confirm the effect of ARG1 overexpression in colon cancer cells on metastatic colonization in vivo, *Arg1* OE and mock control were intrasplenically or intravenously injected into wild-type mice (Fig. 4A). In this study, we confirmed that intra-splenic injection of CT26 cells caused metastatic colonization in the liver, and intravenous injection caused lung metastasis. Furthermore, we could not find the metastatic colonization of lung metastasis by the intra-splenic injection of CT26 cells and liver metastasis by the intra-venous injection at least within 14 days after the inoculations. HPLC analysis indicated that the serum l-arginine levels of both liver and lung metastatic colonization models with *Arg1* OE were lower than those of the mock control (Fig. 4B). Arginase activities were increased in both liver and lung metastatic colonization models with *Arg1* OE compared with the mock control (Fig. 4C). Furthermore, metastatic colonization of *Arg1* OE in the liver as well as in the lung was significantly enhanced compared with the mock control (Fig. 4D–G). In this study, we further evaluated the *Arg1* gene expression levels in the metastatic liver tissues of CT26-bearing mice. As a result, we found that arginase-1 gene expression levels of total liver cells from *Arg1* OE CT26 cell-inoculated mice were much higher than that of mock control cell-inoculated mice. Furthermore, we confirmed that arginase-1 gene expression levels of GFP^+^CD45^−^ tumor cells from *Arg1* OE CT26 cell-inoculated mice were higher compared to the mock control from mice (Additional file 3: Fig. S3). From these data, we speculated that ARG1 overexpression of CT26 colon cancer cells in tumor tissues was possibly related to the augmentation of the serum arginase activity and metastatic colonization in the mouse model.

### ARG1 is expressed in the malignant tumors of colon cancer patients and is involved in the migration ability of human colon cancer cells

**Fig. 5:**
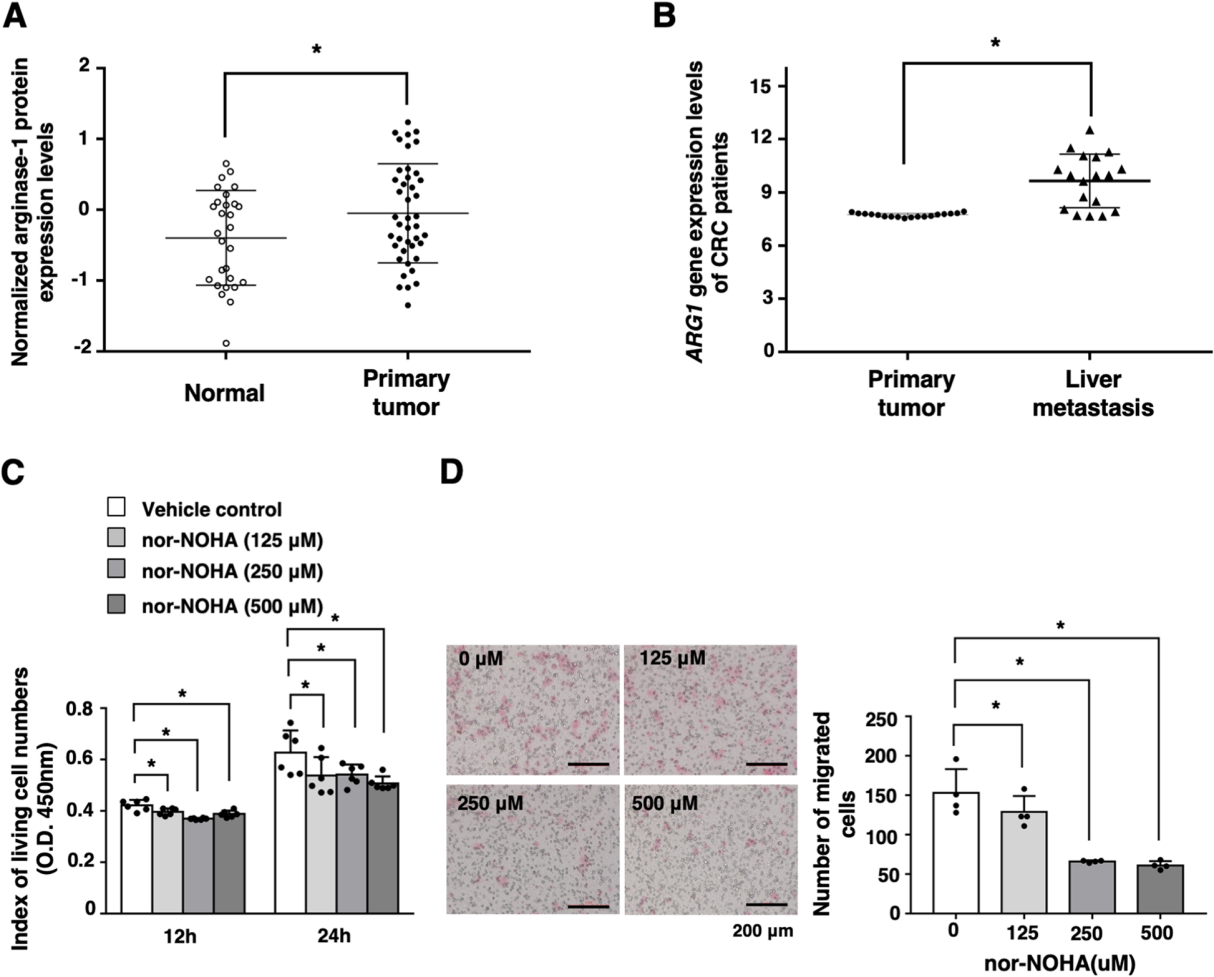
ARG1 activity and gene expression are related to malignancy in human colon cancer. **A** ARG1 protein expression levels in normal (*N* = 28) and tumor (*N* = 42) tissues of CRC patients are shown according to the CPTAC database. *P < 0.05 by unpaired *t*-test. **B** *Arg1* gene expression levels in primary tumor (*N* = 18) and liver metastasis (*N* = 18) of CRC patients were analyzed according to the data from the GEO dataset (GSE14297). *P < 0.05 by unpaired *t*-test. **C** HCT116 cells (5 × 10^3^) were cultured in the absence and presence of nor-NOHA (0, 125, 250, 500 μM). Living cell numbers were evaluated at 12 h and 24 h. The mean and SD values (*n* = 4) are indicated. *P < 0.05 by Student’s *t*-test. **D** HCT116 cells (2 × 10^4^) were cultured in the absence and presence of nor-NOHA (0, 125, 250, 500 μM). Migration ability was evaluated by Transwell assay at 24 h. Representative images are indicated. Bars represent 200 μM. The mean and SD values (*n* = 4) are indicated. *P < 0.05 by Student’s *t*-test

We evaluated serum arginase activities of healthy donors (normal) and CRC patients with informed consent. As a result, the serum arginase activity of CRC patients was higher than those of healthy donors (Additional file 4: Fig. S4). We further investigated whether ARG1 expression levels were related to the tumorigenesis of CRC patients by using published datasets (CPTAC, PDC000116； GEO, GSE14297). The ARG1 protein expression levels of primary tumor were high compared with the normal tissues of CRC patients (Fig. 5A). Moreover, ARG1 gene expression in liver metastasis was significantly higher than in primary tumors of CRC patients (Fig. 5B). Then, we evaluated the effects of ARG1 on the malignancy of human colon cancer cells. Treatment with nor-NOHA significantly suppressed the living cell numbers of HCT116 human colon cancer cells in vitro (Fig. 5C). Inhibition of arginase significantly reduced the migration ability of HCT116 cells in vitro (Fig. 5D). These data suggest that ARG1 expression in colorectal tumors is associated with the recurrence of colon cancer cells, including liver metastasis and ARG1 activity may be related to the living cell numbers and migration of colon cancer cells in humans.

## Discussion

CRC, which is a common cancer worldwide, has a high recurrence rate after surgery and the subsequent occurrence of metastasis that remains to be resolved [1–3]. In this study, we revealed that *Arg1* overexpression significantly promoted the metastatic colonization of colon cancer cells in tumor-bearing mouse models. In contrast, the administration of an arginase inhibitor, nor-NOHA, suppressed the migration ability of colon cancer cells in vitro and metastatic colonization in vivo. Therefore, we speculated that the blockade of ARG1 associated with this malignant transformation might be a promising strategy for the treatment of colon cancer patients.

Liver metastasis of CRC is significantly correlated with poor prognosis and accounts for approximately 50% of cancer-related deaths [22]. Therefore, an investigation of the precise mechanism involved in this malignancy is required for more effective treatment of patients with CRC. Many studies have been conducted to elucidate the control mechanism of cancer recurrence and metastasis. TGF-β produced by immature bone marrow-derived cells provides an environment for the infiltration and metastasis of cancer cells. Myeloid-derived suppressor cells (MDSCs) that have vigorous immuno-suppressive activity have a high expression level of ARG1 [12–14]. Furthermore, many researchers revealed that ARG1-expressing MDSCs induced in a tumor-bearing state were related to the tumorigenesis of colon cancers, and the blockade of this immunosuppressive function showed anti-tumor effects, indicating promising targets for the treatment of CRC patients [24–26]. Recently, we revealed that ARG1 was activated in murine bone marrow-derived dendritic cells and human monocyte-derived dendritic cells to suppress the induction of anti-tumor effector T cells in an IL-6-dependent manner [10, 16]. Furthermore, we constructed a liver metastatic colonization model to examine the relationship between the formation of liver metastases in CRC cells and the host immune system. It was discovered that IL-6 produced in the tumor microenvironment promotes the formation of metastatic lesions in CRC cells [23]. In this study, we confirmed that injection of an arginase inhibitor, nor-NOHA, significantly augmented the infiltration of mature dendritic cells and effector CD8^+^ T cells into tumor tissues as well as the anti-tumor immunity of the liver metastatic colonization model (Additional file 5: Fig. S5). Therefore, our in vivo results indicate that not only does ARG1 activation augment the malignancy of colon cancer cells but also it modulates the tumor microenvironment involved in the dysfunction of host immunity, which facilitates the metastatic colonization of colon cancer cells in the liver. On the basis of these findings, we speculate that inhibition of ARG1 will show effective antitumor effects from two directions in a tumor-bearing host.

The molecular mechanism of ARG1-mediated malignant alteration of colon cancer cells is poorly understood. In this study, we found that ARG1 overexpression was associated with N-cadherin and E-cadherin protein expression levels (Fig. 3G) and the upregulation of *Twist1*, *Twist2*, and Zeb2 gene expressions (Additional file 2: Fig. S2), which have a crucial role in EMT [27, 28]. EMT promotes the migration and metastasis of cancer cells [29] as well as tumor aggressiveness with worse recurrence-free survival [30, 31]. A previous paper revealed that overexpression of ARG1 led to a significant increase in the expression of Vimentin, N-cadherin, and *β*-catenin both at protein and mRNA levels of hepatocellular carcinoma (HCC) [32]. These findings suggest that ARG1 expression may be related to the metastasis of colon cancer cells as well as HCC through promoting the EMT process. Therefore, we speculated that ARG1 possibly promoted the migration ability and metastatic colonization of colon cancer cells through the augmentation of EMT pathways in this study.

Previous studies have reported that ARG1 is expressed in many types of cancers such as breast cancer and liver cancer [33–35]. However, an association between ARG1 and CRC has not been fully reported. In this study, we demonstrated that ARG1 activity is related to the metastatic colonization of colon cancer cells in the liver and lung tissue of mice. In addition, we found that serum arginase activity and l-arginine levels were correlated with metastatic colonization in our mouse models. In this study, we revealed that blockade of arginase activity and l-arginine supplementation significantly reduced metastatic colonization of CT26 cells in the liver of the mice (Additional file 1: Fig. S1). Furthermore, we confirmed that arginase activity is involved in the migration ability of human colon cancer cells, and ARG1 is expressed in the malignant tumors of colon cancer patients, suggesting that they might be not only promising biomarkers but also therapeutic targets for advanced CRC patients.

## Conclusion

Our data suggest that activation of ARG1 promotes the migration, colonization, and metastasis of colon cancer cells in tumor-bearing hosts. Blockade of ARG1 activity may suppress the malignant alteration of CRC cells. These results suggest that the ARG1-mediated arginine metabolism may be a new target to regulate the liver metastasis of colon cancer cells in CRC patients.

## Supplementary Information

**Additional file 1: Fig. S1.** Supplementation of L-arginine significantly suppresses the liver metastatic colonization of colon cancer cells. GFP-transfected CT26 murine colon cancer cells (2 × 10^5^) were intrasplenically inoculated into wild-type BALB/c mice (day 0). Then, L-arginine (500 mg/kg) or PBS was injected intraperitoneally on days 5, 7, 9, 11, and 13. Liver tissues of the CT26 cell-inoculated mice were collected on day 14. **A,** Metastatic colonization in liver tissue of mice injected with L-arginine or PBS was evaluated using an *in vivo* imaging system at day 14. Representative images of GFP-expressing CT26 cell-bearing livers are shown. Photon flux ratios were determined from images of liver metastatic colonization model mice (n = 6). *P < 0.05 by Student’s t-test. **B,** HE staining of liver tissue was performed at 14 days after inoculation. Bars in the images represent 500 μm. Ratios of tumor area relative to total liver tissue area were calculated by ImageJ software. Mean values and SDs from four independent mice are shown. *P < 0.05 by Student’s t-test.

**Additional file 2: Fig. S2.** Expression levels of EMT-related genes are augmented in *Arg1*-overexpressing CT26 cells compared to the mock control cells. GFP-transfected CT26 mock control and CT26 *Arg1* OE cells were established using pMX-IRES-GFP vector. **A,** Gene expression levels of *Twist1*, *Twist2*, *Zeb2, and Actb* were investigated by qPCR. Relative gene expression levels of *Twist1*, *Twist2*, and *Zeb2* in *Arg1* OE cells to the mock control cells were evaluated and mean values and SDs (n = 4) are indicated. *P < 0.05 by Student’s t-test.

**Additional file 3: Fig. S3.** ARG1 gene expression levels in the liver tissues of *Arg1* OE-inoculated mice are higher than those in the mock control. GFP-transfected CT26 murine colon cancer cells (2 × 10^5^) were intrasplenically inoculated into wild-type BALB/c mice (day 0). Liver tissues of the CT26 cell-inoculated mice were collected on day 14. GFP^+^CD45^-^ CT26 cells were isolated ed from the collagenase-treated liver tissues by the cell sorter. Relative *Arg1* gene expression levels of the total liver cells and GFP^+^CD45^-^ CT26 cells from *Arg1* OE- or the mock control-inoculated mice were evaluated by qPCR. Mean and SD values (n = 4) are indicated. *P < 0.05 by Student’s t-test.

**Additional file 4: Fig. S4.** Arginase activity in the serum of CRC patients is higher than those of healthy donors. Arginase activities of sera from CRC patients and healthy donors (Normal) were evaluated by EIA. Mean values and SDs (normal = 11, CRC patient = 9) are shown. *P < 0.05 by Student’s t-test.

**Additional file 5: Fig. S5.** Inhibition of arginase activity significantly augments anti-tumor immunity in the liver metastatic colonization model. GFP-transfected CT26 murine colon cancer cells (2 × 10^5^) were intrasplenically inoculated into wild-type BALB/c mice (day 0). Then, nor-NOHA (20 mg/kg) was injected intraperitoneally on days 5, 7, 9, 11, and 13. Liver tissues of the CT26 cell-inoculated mice were collected on day 14. **A,** Tumor-infiltrating CD11c^+^ DCs and CD3^+^ T cells were evaluated by IHC. Representative images are shown. Bars in the images represent 200 mm. **B,** Mature DCs and effector memory CD8^+^ T cells in the liver were evaluated by flow cytometry. Mean and SD values (n = 4) are indicated. *P < 0.05 by Student’s t-test. **C,** Perforin- or granzyme B-expressing CD8^+^ T cells in the liver were evaluated by flow cytometry. Representative images; mean and SD values (n = 4) of the percentages or ΔMFIs are indicated. *P < 0.05 by Student’s t-test.

## Data Availability

Raw proteomics data of ARG1 protein expression in patients with colon cancer and normal solid tissue are hosted by the CPTAC data portal (https://cptac-dataportal.georgetown.edu/cptacPublic/). *ARG1* RNA expression profiles of primary colorectal cancers with liver metastases of the same patient were obtained from the Gene Expression Omnibus (GEO) at GSE14297. The datasets used and/or analyzed during the current study are available from the corresponding author upon reasonable request.

## Abbreviations

ARG1: Arginase-1
CRC: Colorectal cancer
EMT: Epithelial-mesenchymal transition
MDSC: Myeloid-derived suppressor cell
