# Supplementary figures and images for: Arginase-1 inhibition reduces migration ability and metastatic colonization of colon cancer cells
Source: Cancer Metab. 2023 Jan 13;11:1. doi: 10.1186/s40170-022-00301-z (PMC9838026; doi:10.1186/s40170-022-00301-z)

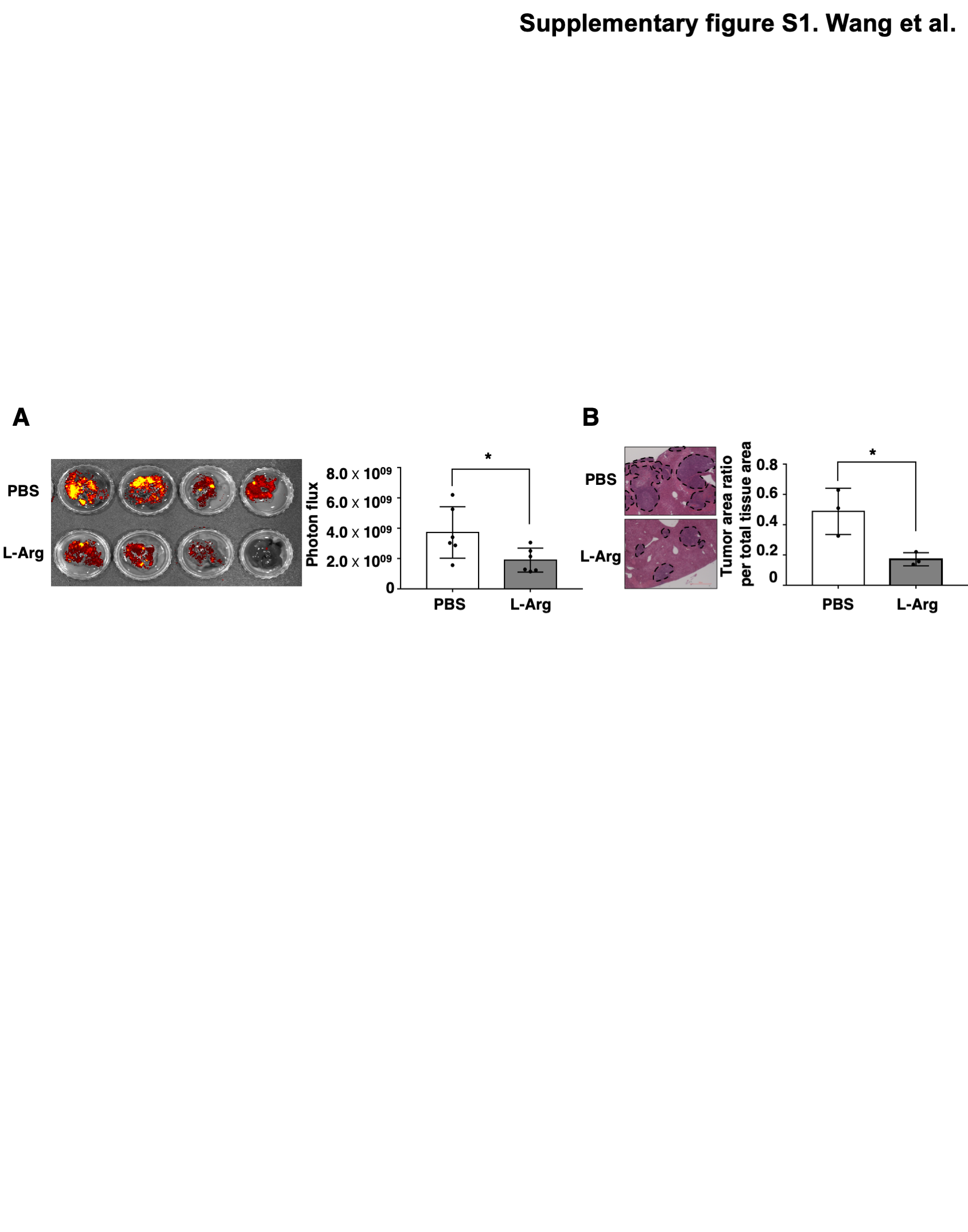

Supplement: Supplementary file 1 — Additional file 1: Fig. S1. Supplementation of L-arginine significantly suppresses the liver metastatic colonization of colon cancer cells. GFP-transfected CT26 murine colon cancer cells (2 × 10^5^) were intrasplenically inoculated into wild-type BALB/c mice (day 0). Then, L-arginine (500 mg/kg) or PBS was injected intraperitoneally on days 5, 7, 9, 11, and 13. Liver tissues of the CT26 cell-inoculated mice were collected on day 14. A, Metastatic colonization in liver tissue of mice injected with L-arginine or PBS was evaluated using an in vivo imaging system at day 14. Representative images of GFP-expressing CT26 cell-bearing livers are shown. Photon flux ratios were determined from images of liver metastatic colonization model mice (n = 6). *P < 0.05 by Student’s t-test. B, HE staining of liver tissue was performed at 14 days after inoculation. Bars in the images represent 500 μm. Ratios of tumor area relative to total liver tissue area were calculated by ImageJ software. Mean values and SDs from four independent mice are shown. *P < 0.05 by Student’s t-test. [file 40170_2022_301_MOESM1_ESM.tiff]

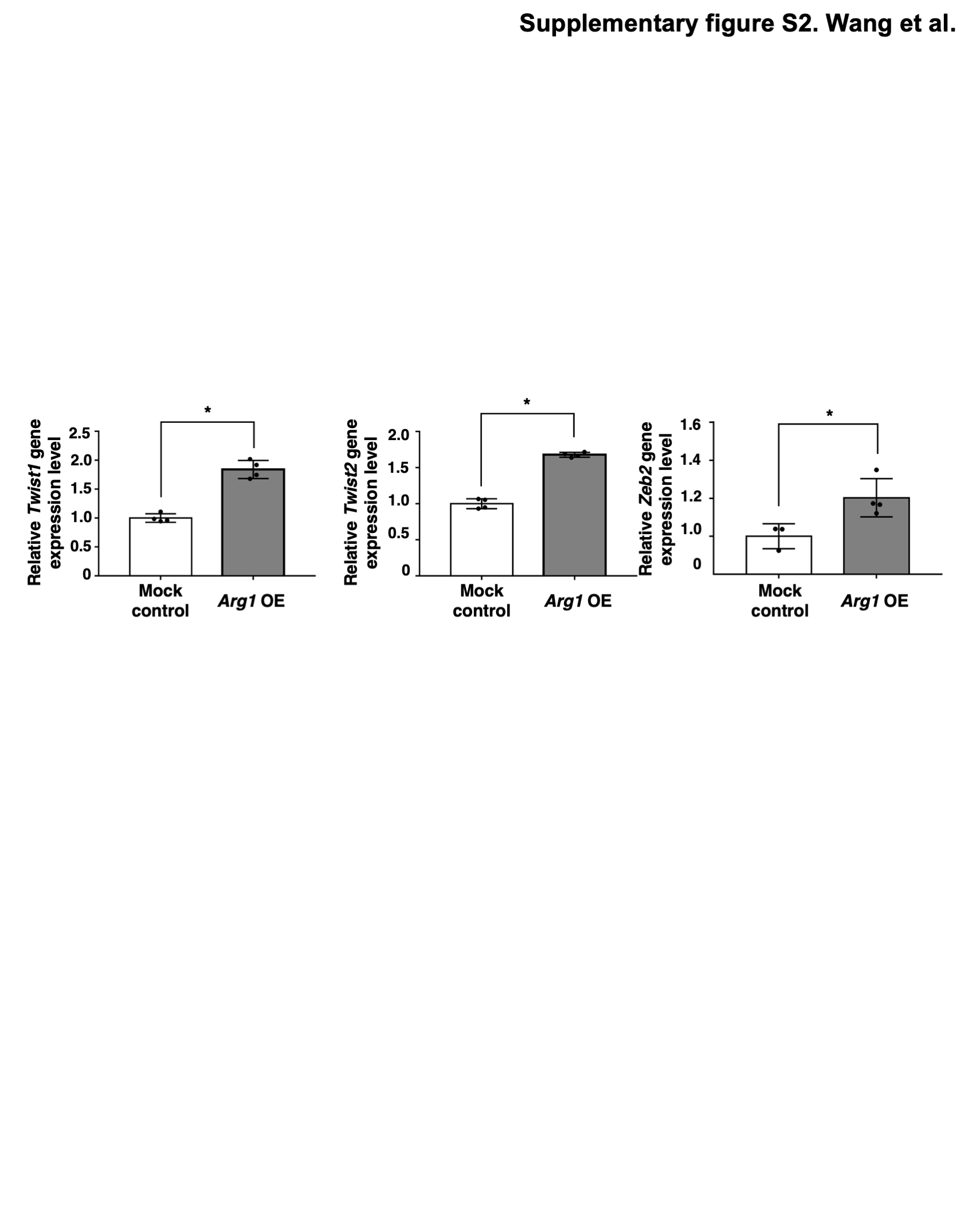

Supplement: Supplementary file 2 — Additional file 2: Fig. S2. Expression levels of EMT-related genes are augmented in Arg1-overexpressing CT26 cells compared to the mock control cells. GFP-transfected CT26 mock control and CT26 Arg1 OE cells were established using pMX-IRES-GFP vector. A, Gene expression levels of Twist1, Twist2, Zeb2, and Actb were investigated by qPCR. Relative gene expression levels of Twist1, Twist2, and Zeb2 in Arg1 OE cells to the mock control cells were evaluated and mean values and SDs (n = 4) are indicated. *P < 0.05 by Student’s t-test. [file 40170_2022_301_MOESM2_ESM.tiff]

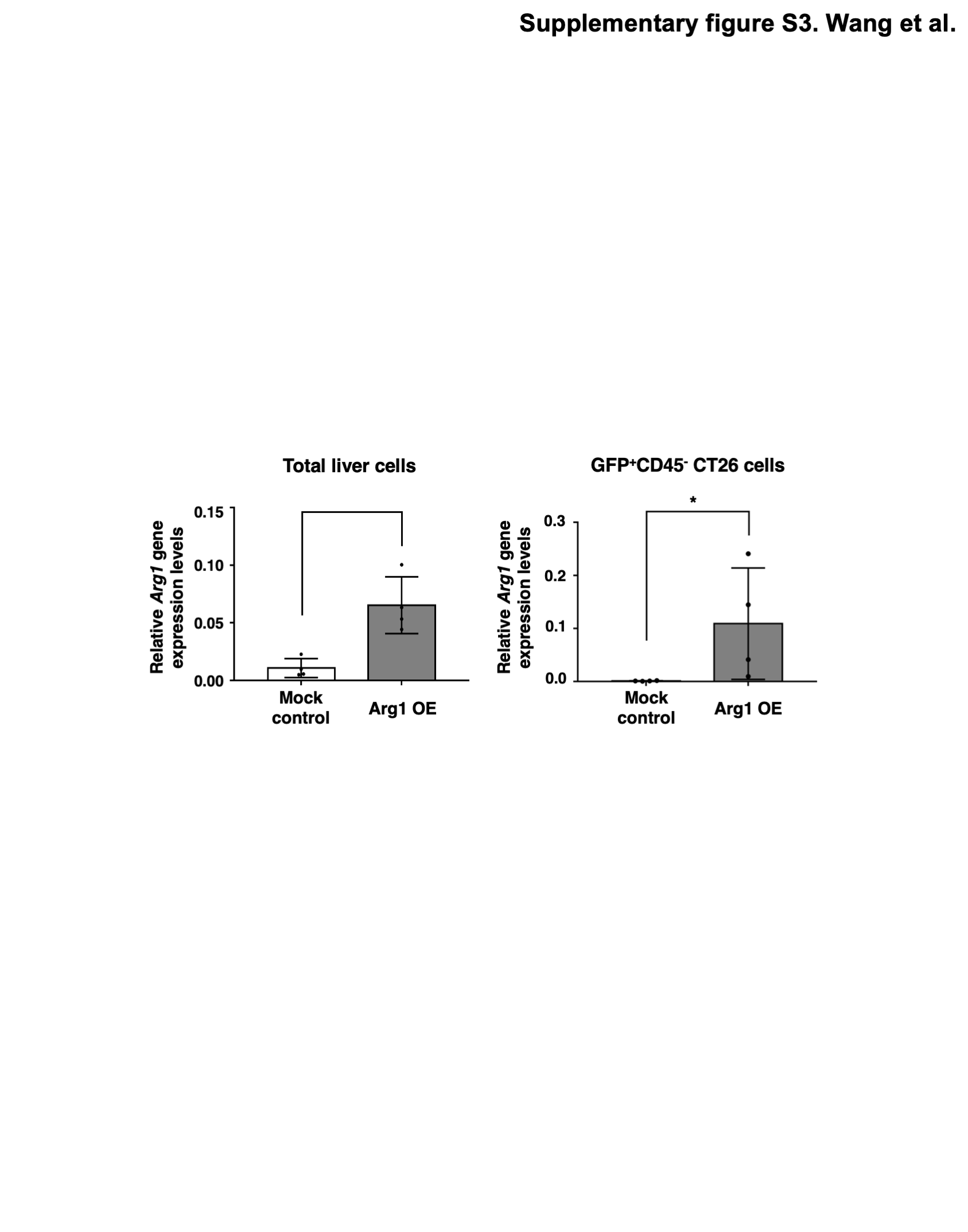

Supplement: Supplementary file 3 — Additional file 3: Fig. S3. ARG1 gene expression levels in the liver tissues of Arg1 OE-inoculated mice are higher than those in the mock control. GFP-transfected CT26 murine colon cancer cells (2 × 10^5^) were intrasplenically inoculated into wild-type BALB/c mice (day 0). Liver tissues of the CT26 cell-inoculated mice were collected on day 14. GFP+CD45- CT26 cells were isolated ed from the collagenase-treated liver tissues by the cell sorter. Relative Arg1 gene expression levels of the total liver cells and GFP+CD45- CT26 cells from Arg1 OE- or the mock control-inoculated mice were evaluated by qPCR. Mean and SD values (n = 4) are indicated. *P < 0.05 by Student’s t-test. [file 40170_2022_301_MOESM3_ESM.tiff]

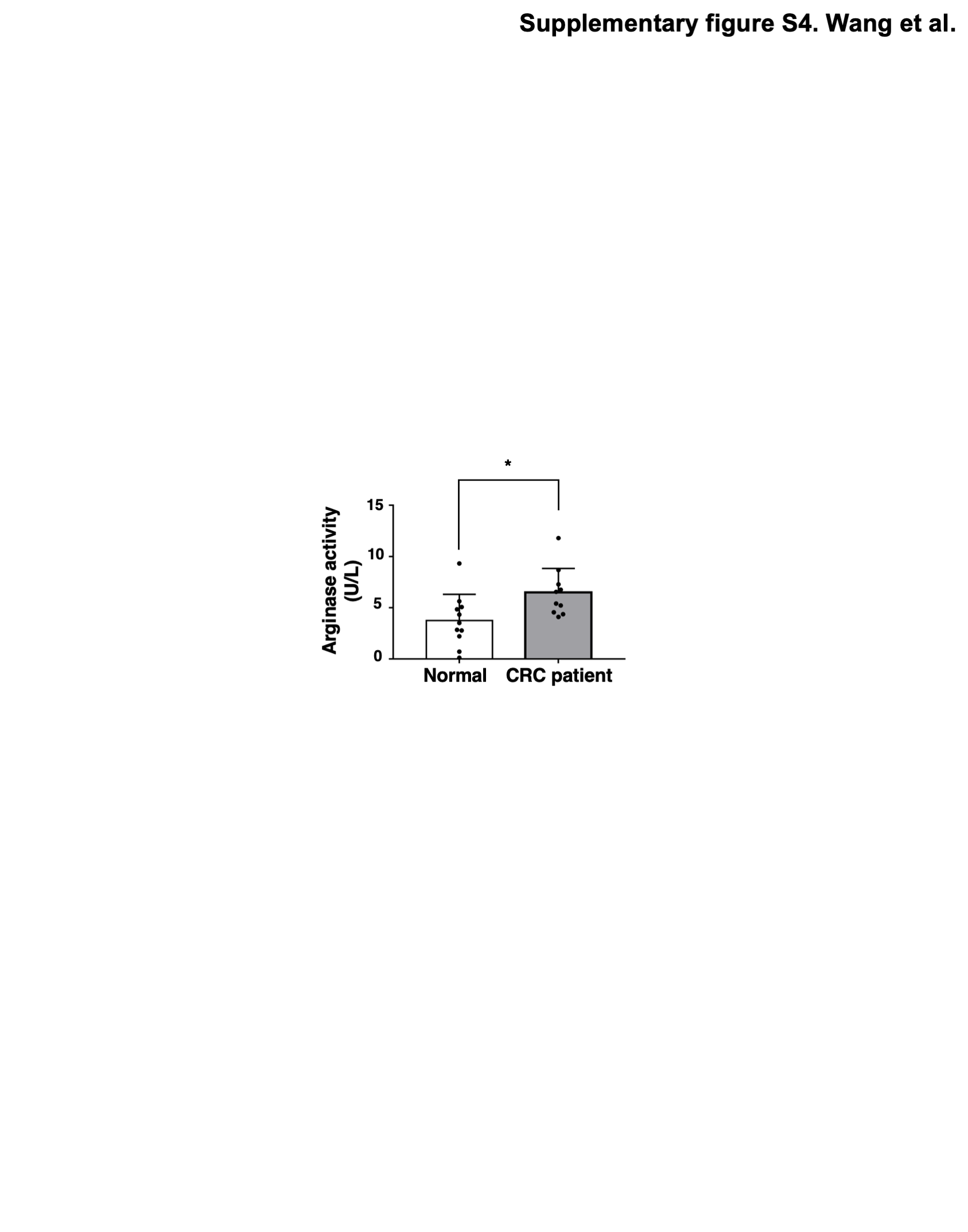

Supplement: Supplementary file 4 — Additional file 4: Fig. S4. Arginase activity in the serum of CRC patients is higher than those of healthy donors. Arginase activities of sera from CRC patients and healthy donors (Normal) were evaluated by EIA. Mean values and SDs (normal = 11, CRC patient = 9) are shown. *P < 0.05 by Student’s t-test. [file 40170_2022_301_MOESM4_ESM.tiff]

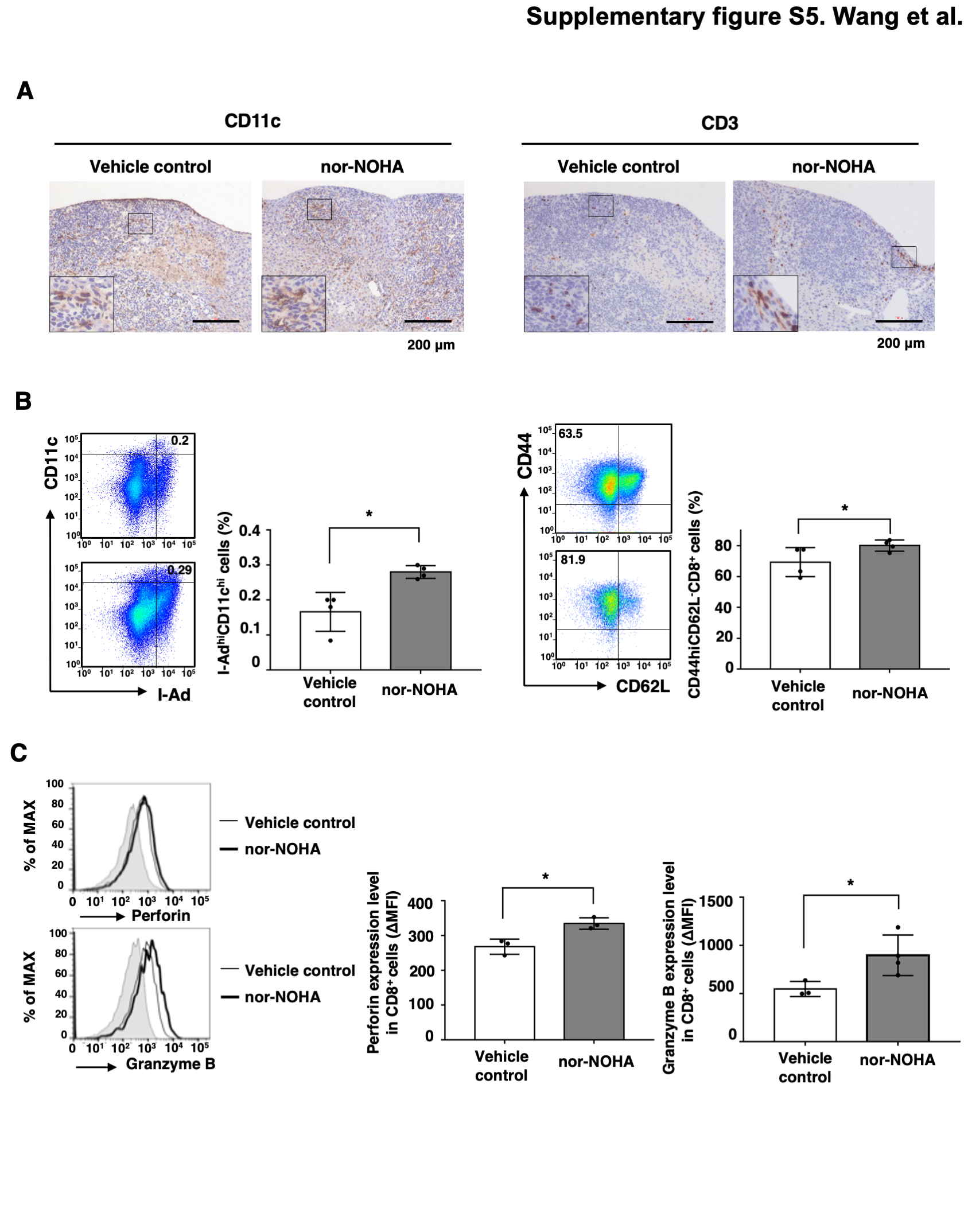

Supplement: Supplementary file 5 — Additional file 5: Fig. S5. Inhibition of arginase activity significantly augments anti-tumor immunity in the liver metastatic colonization model. GFP-transfected CT26 murine colon cancer cells (2 × 10^5^) were intrasplenically inoculated into wild-type BALB/c mice (day 0). Then, nor-NOHA (20 mg/kg) was injected intraperitoneally on days 5, 7, 9, 11, and 13. Liver tissues of the CT26 cell-inoculated mice were collected on day 14. A, Tumor-infiltrating CD11c+ DCs and CD3+ T cells were evaluated by IHC. Representative images are shown. Bars in the images represent 200 mm. B, Mature DCs and effector memory CD8+ T cells in the liver were evaluated by flow cytometry. Mean and SD values (n = 4) are indicated. *P < 0.05 by Student’s t-test. C, Perforin- or granzyme B-expressing CD8+ T cells in the liver were evaluated by flow cytometry. Representative images; mean and SD values (n = 4) of the percentages or ΔMFIs are indicated. *P < 0.05 by Student’s t-test. [file 40170_2022_301_MOESM5_ESM.tiff]
